# A Statistical Analysis of the Robustness of Alternate Genetic Coding Tables

**DOI:** 10.3390/ijms9050679

**Published:** 2008-05-02

**Authors:** Mehmet Levent Kurnaz, Tugce Bilgin, Isil Aksan Kurnaz

**Affiliations:** 1 Physics Department, Bogazici University Bebek, 34342 Istanbul, Turkey. E-mail: levent.kurnaz@boun.edu.tr; 2 Department of Genetics and Bioengineering, Yeditepe University Kayisdagi, 34755 Istanbul, Turkey. E-mail: tugce_bilgin@yahoo.co.uk. E-mail: iakurnaz@yeditepe.edu.tr

**Keywords:** genetic code, evolution, robustness, statistical analysis

## Abstract

The rules that specify how the information contained in DNA is translated into amino acid “language” during protein synthesis are called “the genetic code”, commonly called the “Standard” or “Universal” Genetic Code Table. As a matter of fact, this coding table is not at all “universal”: in addition to different genetic code tables used by different organisms, even within the same organism the nuclear and mitochondrial genes may be subject to two different coding tables. Results In an attempt to understand the advantages and disadvantages these coding tables may bring to an organism, we have decided to analyze various coding tables on genes subject to mutations, and have estimated how these genes “survive” over generations. We have used this as indicative of the “evolutionary” success of that particular coding table. We find that the “standard” genetic code is not actually the most robust of all coding tables, and interestingly, Flatworm Mitochondrial Code (FMC) appears to be the highest ranking coding table given our assumptions. Conclusions It is commonly hypothesized that the more robust a genetic code, the better suited it is for maintenance of the genome. Our study shows that, given the assumptions in our model, Standard Genetic Code is quite poor when compared to other alternate code tables in terms of robustness. This brings about the question of why Standard Code has been so widely accepted by a wider variety of organisms instead of FMC, which needs to be addressed for a thorough understanding of genetic code evolution.

## 1. Introduction

How the genetic code evolved has been a matter of interest for many researchers over the past decades – Crick [1] had postulated the coevolution and frozen accident hypotheses, where similar amino acids would end up using similar codons as a result of coevolution of coding tables and genes, and remain “frozen” at an optimum coding that reduces deleterious effects of mutations (reviewed in [2]). One of the important properties of a genetic code is its *robustness* to error, which means that if a mutation occurs in a gene, the amino acid substitution ideally renders a functionally similar protein, thus a robust code reduces the deleterious effects of mutations. Thus one would at first sight assume that the coding table that has been adopted by a wider range of organisms would appear more robust, which has been the basic premise behind our analysis.

**Figure 1.**
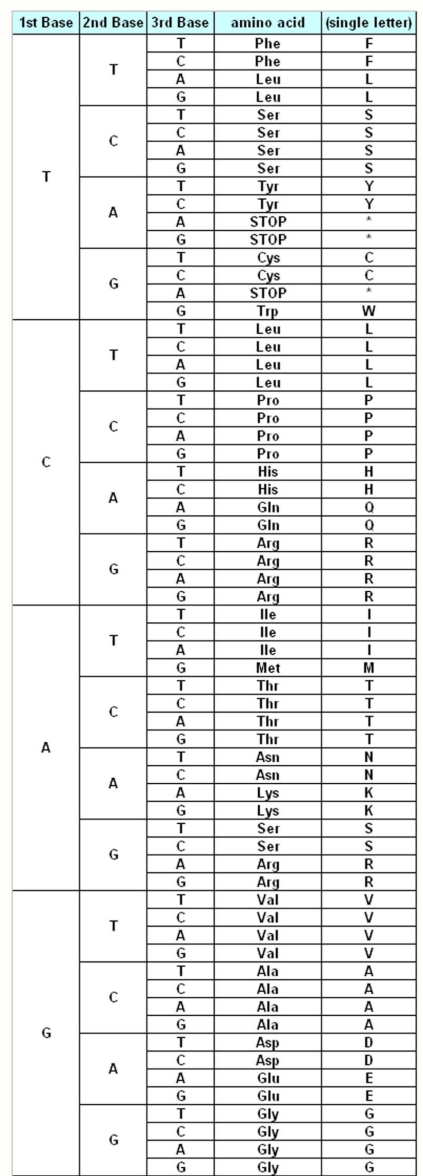
The “Universal Genetic Code Table”, adopted from Introduction to Biology, Campbell and Reece (6^th^ Ed, 2002). START codon (AUG) encodes for Methionine (Met, M), and the three STOP codons are indicated (UAA, UAG, UGA).

The genetic information about the individuals is stored in the DNA, which make up the genes. DNA is made up of different monomers, or nucleotides, containing one of the four heterocyclic bases: adenine (A), guanine (G), cytosine (C) and thymine (T). Genes use triplet codes (“codons”) to translate the information into proteins – each of the 20 amino acids is coded by three-base combinations (Figure 1). The Genetic Code Tables summarize how this codon assignment is made, saving three codons to signal “STOP” for protein synthesis machinery, i.e. 20 amino acids are encoded by 61 different codons. There are various exceptions to this Universal/Standard Coding Table, however – for instance vertebrate and invertebrate mitochondria use different coding tables for their own genes, as do Ciliates (Table 1). The alternate coding tables are believed to have arisen from the evolution of the standard genetic code through codon reassignments, and most studies on possible mechanisms of this evolution start out by the assumption that the changes resulting in codon reassignment would be strongly disadvantegous and consequently get eliminated from the system [3,4]. Using a similar assumption, our present study aims to compare the possible “evolutionary” advantages of these different genetic codes in terms of robustness and resilience to mutations.

In our study, genes or “individuals” are represented by bit-strings which are 32 bits long and are initially set to zero. Each bit represents a given age or generation: as the individual reproduces we move down on the bit-string. Bits which are set to zero represent that no deleterious mutations happened at that age. However, if a bit is set to one, it means that the individual suffers a severe mutation at that generation and its probability of survival or viability is compromised. This is based on previous reports that a lineage of organisms where mutations result in chemically conserved amino acid substitutions may actually have higher survivability as compared to those with a less conservative code [5,6], and according to the error-minimization hypotheses, the universal or standard genetic code has evolved an inverse relationship between the severity and frequency of these alterations [5,7]. We have previously used this model to show the optimal number of amino acids that could be encoded by 64 codons without affecting survivability of populations due to deleterious mutations [8]. In this initial set of analyses, we make two simplified assumptions: first of all, we assumed that nucleotide substitutions occur at similar frequencies (current work is integrating unequal substitution rates; unpublished data). Secondly, we assume that any change in amino acid composition would be deleterious, hence we do not incorporate similarity matrices for the purposes of simplification in this present study (ongoing work is incorporating BLOSUM matrices, without significant alterations in our findings; unpublished data).

Here, we have analyzed a variety of genes from different organisms against 12 different coding tables. Our analysis is based on the fundamental assumption that if the gene being analyzed is coding for an essential component of the cells in that organism, such as integrity, metabolism, or replication of DNA, it becomes very important that the gene remain functional in order for the organism to survive. The underlying assumption is that the mutations which render this particular gene completely inactive would mean that the individual would not “survive” [8]. Thus, over a number of generations, we could analyze what the survivability outcome is with respect to the entire population – since the mutation is considered in the light of the particular coding table analyzed, the better the survivability, the more robust the coding table (for details, see Methods).

Our results show that the “Universal Genetic Code” is actually sub-optimal in terms of robustness in this simplified analysis, and FMC appears to function significantly better in protecting the genes against mutations described in our study. For ciliate and hexamite representative genes only, The Ciliate, Dasycladacean and Hexamita Nuclear Code (CDH) appears to be on a par with FMC in terms of robustness, while Yeast (YMC) and Vertebrate Mitochondrial (VMC) codes are unsuccessful. It is rather puzzling that a relatively poor-performing Standard Code Table has been adopted by such a wide variety of organisms, and further analyses need to be performed in order to thoroughly understand the nature of the genetic codes. It should be noted, however, that differences in nucleotide substitution rates, and various amino acid substitution matrices should be incorporated in a larger study (work still ongoing), however our preliminary results indicate that the overall profile of robustness among coding tables do not significantly change (unpublished data). It should also be noted that the initial environment when the coding tables were still diverging was significantly different than the conditions today, and responses of populations to mutations could be similarly different, and some mutations could perhaps have been allowed. This study should therefore be further improved in order to consider many aspects, but should be seen as an initial step towards such an improvement.

## 2. Results and Discussion

A previous study [8] had studied how an *in silico* population survived over generations, by calculating the probability of “survival” upon random mutations of an essential gene – the so-called “human cytokine” gene – where a mutation that renders the protein non-functional resulted in death of the organism. The results in that study were rather interesting, showing that the optimum number of amino acids that could be encoded by the coding table that resulted in optimum survival of the population was indeed 22, rather than the 20 amino acids normally found in the Universal Coding Table (Figure 1, [8]).

This result by itself was rather intriguing, taken together with the fact that some genetic code reassignments and expansions of the coding tables are still ongoing [9]. This has led us to the question of the performance of the alternate coding tables. Using the same statistical analysis, we wished to address whether the universal coding table was even slightly more robust than the alternate tables in terms of resilience against mutations.

To that end, we have analyzed several genes that are either ubiquitous or important for the integrity and functionality of cells of the body in humans and primates; such as actins, which are highly conserved proteins involved in cell motility and maintenance of the cytoskeleton, and tubulin isoforms, which are the main components of the microtubular network and functionally important for cellular integrity as well as mitosis (see Table 2 for a comprehensive list of genes and NCBI accession numbers).

**Figure 2.**
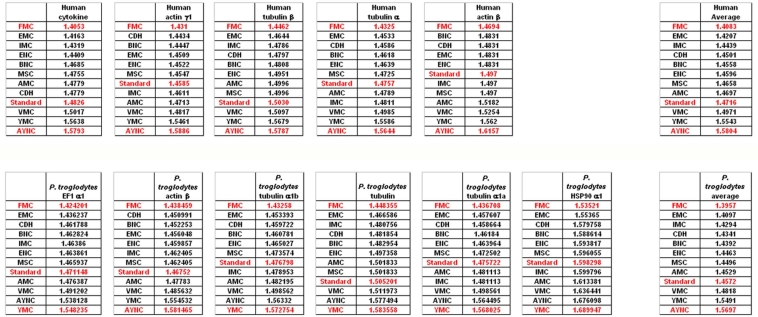
Comparison of various coding tables with respect to robustness for selected human and primate genes that are normally subject to Standard Genetic Code. “Average” genes represent hypothetical and idealized genes constructed using average codon usage frequencies (see Methods); accession numbers of the genes are listed in Table 2. Coding table abbreviations are given in Table 1.

When we have analyzed the effects of various coding tables on these genes as described in Methods section, we have observed that the Standard (or Universal) Code has performed significantly worse than many other coding tables, ranking between 6^th^ and 9^th^ among all 12 coding tables tested (Figure 2). In order to assess whether this low performance was simply due to a bias of these genes with respect to the average codon usage frequencies in the corresponding organisms, we have also generated so-called “average” genes, which are randomly representative of codon usage frequencies previously determined for those particular genomes (http://www.kazusa.or.jp) (Figure 2). The Standard Coding Table provided poor resilience towards mutations even in this hypothetical gene, whereas FMC performed much better in almost all of the human genes tested.

**Figure 3.**
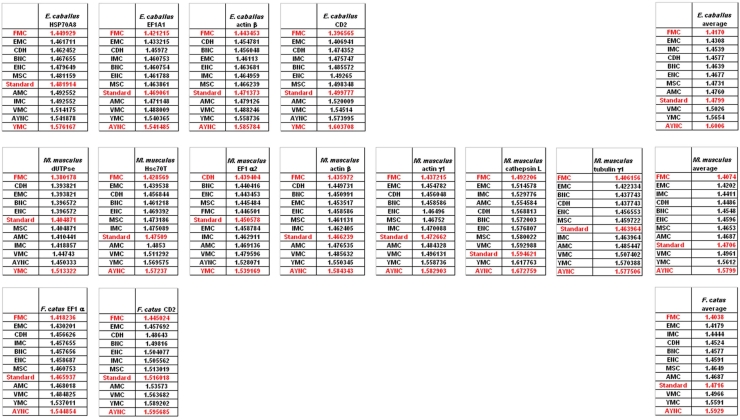
Comparison of various coding tables with respect to robustness for selected genes from cat, horse and mouse that are normally subject to Standard Genetic Code. “Average” genes represent hypothetical and idealized genes constructed using average codon usage frequencies (see Methods); accession numbers of the genes are listed in Table 2. Coding table abbreviations are given in Table 1.

Of course, this could have been due to something peculiar about primate genes and requirements of these genomes. Thus, we have decided to analyze genes from representative mammals, essentially horse, cat and mouse (Figure 3). The ranking of the Standard Code among all other tables tested was pretty variable in mouse, albeit still low in terms of performance. Similar to primate genes, we wanted to check whether codon usage frequencies of these genomes could in fact have affected the analyses, and constructed hypothetical “average” genes also for these genomes. The Standard Code was still poor-performing, ranking 9^th^ in all three organisms (Figure 3).

One of the better-performing nuclear coding tables in this analytical scheme was the CDH code, usually ranking between 2^nd^ and 3^rd^ positions – thus we wanted to address whether this nuclear code would still perform better when genes normally subject to this coding table were analyzed. To that end, we have used representative genes from 4 different species, all of which are involved in DNA replication machinery, and also constructed two “average” genes based on codon usage frequencies (Table 2). In all of the 4 genes selected, FMC was still a better-performing coding table, ranking the first in “survivability”, with one exception being the Hexamite Elongation Factor 1 (EF1) gene (Figure 3). Interestingly, Standard Code also performed slightly better for these genes, ranking between 4^th^ and 6^th^ among the 12 coding tables (Figure 3).

**Figure 4.**
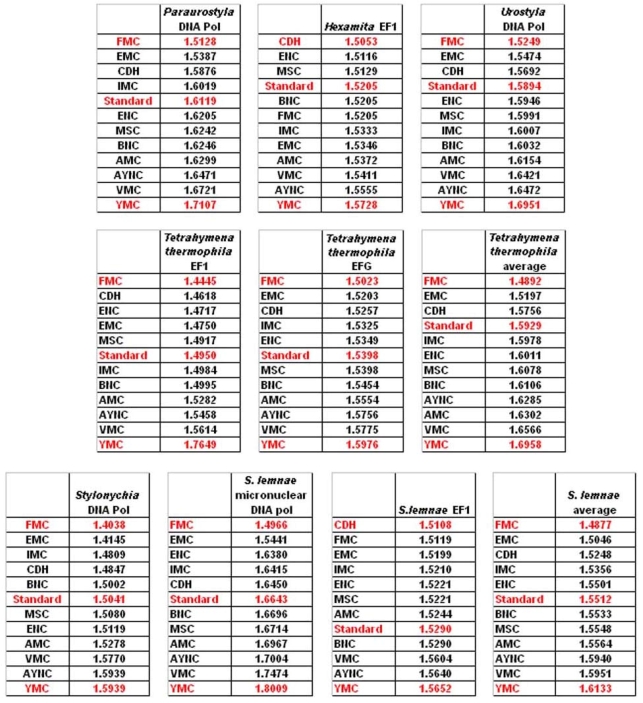
Comparison of various coding tables with respect to robustness for selected genes that are normally subject to CDH Coding Table. “Average” genes represent hypothetical and idealized genes constructed using average codon usage frequencies (see Methods); accession numbers of the genes are listed in Table 2. Coding table abbreviations are given in Table 1.

We have observed similar results in our analyses of ENC and MSC tables (data not shown): essentially, FMC was the best scoring table among all, with CDH in the top 4 in all of the assays (Figure 4).

**Figure 5.**
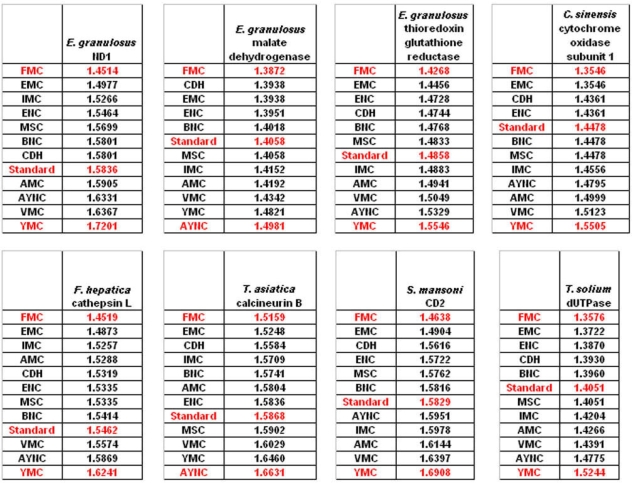
Comparison of various coding tables with respect to robustness for selected genes that are normally subject to FMC Coding Table. “Average” genes represent hypothetical and idealized genes constructed using average codon usage frequencies (see Methods); accession numbers of the genes are listed in Table 2. Coding table abbreviations are given in Table 1.

The FMC appearing at the top of the list in most of the analyses was rather intriguing, especially since other mitochondrial coding tables have been usually the worst performing tables in all the analyses so far. Thus, we wanted to initially address the question of how this coding table performed with respect to the genes that normally utilizes this table, namely genes encoded by flatworm mitochondrial DNA. To that end, we have used several different mitochondrial genes from various flatworm species; in order to compare mitochondrial versus nuclear gene performance, and also constructed “average” genes for this table (Table 2). In all mitochondrial genes, FMC appeared as the highest-performing coding table, as with other species and coding tables examined so far (Figure 5). The Standard Code was still suboptimal for these genes, ranking 5^th^-8^th^ among the 12 coding tables tested, while CDH is still a better performer than the Standard Code (Figure 5). For vertebrate or invertebrate mitochondrial tables, the genes encoded by mitochondrial genomes were analyzed, however the overall profile had not changed, with FMC still outperforming the Standard Code (data not shown).

**Figure 6.**
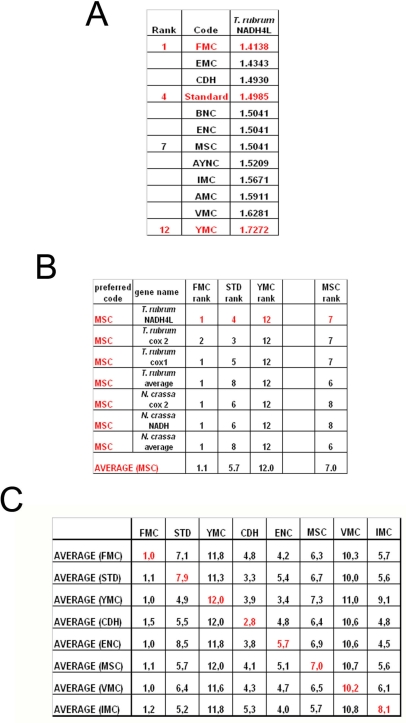
Ranks of coding tables across organisms and genes in terms of robustness and survivability. (A) A representative list of statistical calculations of survivability for a given gene if it were subject to different coding tables. (B) For genes that are normally decoded by one given Code Table, the average rank of certain alternate tables in terms of performance in survivability (projection of robustness). (C) A summary of performance comparison of all the coding tables tested with respect to one another. The rows show averages of all the genes analyzed for that particular coding scheme (ie, AVERAGE(FMC) means average ranks for genes that are normally subject to FMC), while the columns indicate what would have happened if alternative coding schemes were adopted.

To summarize all these scores in one table, we have taken the rankings of all coding tables used for each gene (Figure 6a). Afterwards, for all genes analyzed from that particular table (for instance MSC), these ranks were summed up and their averages were calculated (Figure 6b). When this was calculated for all the genes tested from all the different coding tables, we have organized our data in a tabulated form (Figure 6c). As can be seen, quite unexpectedly, Standard coding table ranged in performance ranking from 4.9 to 7.9, which was rather poor when compared to the FMC table, which was almost always 1st in the genes tested across species (Figure 3c). The worst performing table in almost all the cases was yet another mitochondrial table, YMC, which indicates that the results are not correlated with whether the genes are encoded by nuclear or mitochondrial genomes, or by mutations rates thereof. VMC was slightly better than YMC, but interestingly, IMC was closer to the Standard Code than to VMC (Figure 3c).

## 3. Conclusions

In this paper, we have used statistical analysis to investigate the optimality of alternate genetic coding tables on the “survivability” of genes representative of different organisms. This analysis simply calculates the probability of maintenance of a functional protein after generations of single-base substitutions in a given gene, depending on whether the mutation is silent, missense or nonsense in that particular coding table used (see Methods). Our results indicate that the Standard, or “Universal”, Genetic Code is actually one of the lower performance tables in terms of tolerating mutations and rendering another functional protein upon genetic substitutions. The best success rates were obtained, surprisingly, with FMC for all the organisms tested, which was not paralleled in either the VMC or other mitochondrial coding tables analyzed (Figure 6)

Interestingly, the CDH does in fact give the maximum score for the *Hexamita* gene, EF1, which in fact does use this very coding table, and CDH performs significantly better than the Standard Code for other ciliate and *Hexamita* genes that we have analyzed. This may imply that indeed CDH might be evolutionarily more adapted to the organism and the environment against any possible mutations (see below).

There have been many studies trying to explain the presence of alternate genetic codes, or indeed why the standard genetic code is still undergoing reassignments of codons [5,7,9,10], which required modification of the so-called “frozen-accident” theory [1]. These changes in alternate code tables are believed to have stemmed from reassignments of codons of the Standard Genetic Code, and not from ancestral lineages of alternate coding tables [10]. Then one could imagine a situation where newly evolved codes due to evolution may indeed by better suited for certain organisms and certain conditions, in line with our data on FMC, nevertheless this still fails to explain why FMC and not any other mitochondrial code?

This could in part be explained by the nature of codon reassignments in these particular code tables: when IMC, VMC, YMC, and FMC are all compared, there are a few common reassignment schemes – UGA that is STOP in the Standard Code is reassigned to Trp in these mitochondrial codes, and AUA that is Ile in the Standard code is assigned to Met in all these codes except for FMC (see Table 1 and Figure 1). However, when the differential reassignments are analyzed, it becomes apparent that there is an entire subset of codons for Leucine (CUU, CUC, CUA and CUG) that has been reassigned to another amino acid, Threonine (Table 1) in YMC table, leaving only two codons still encoding Leu. Also in YMC, the UGA STOP codon has been reassigned to Trp, which would lead to failure to stop translation of certain proteins. Furthermore, to make things even worse for YMC, two codons, CGC and CGA, have been left unassigned (Table 2). Similarly, in the VMC scheme, AGA and AGG codons for Arginine have been reassigned to STOP, which could result in immature termination of translation, thus affecting the performance of this coding table immensely.

When the FMC table was analyzed, however, one can readily observe that the reassignments are relatively “mild” when compared to the other coding tables: the UAA STOP codon has been changed to Tyrosine, reducing one STOP codon, however at the same time increasing the robustness to any mutations to the two Tyrosine codons, UAU and UAC (see Figure 1 and Table 2). The AAA codon for the positively-charged Lys has been rassigned to a polar Asn residue, and the two Arg codons, namely AGA and AGG, have been reassigned to another polar residue, Ser (Table 2). When compared to our results, these changes would appear to have increased the tolerance of this coding table to mutations that affect primary structure of the protein (Figure 5). Of course, one must note that this initial study excludes any similarity matrices for mutations as it is, however recent data indicate that improvement of the calculations based on input from BLOSUM, PAM (Point Accepted Mutations) and other matrices does not significantly alter the ranking profile of these coding tables (Kurnaz and Kurnaz, unpublished data). This could partly be explained by the fact that PAM matrix itself may be a result of the nature of the genetic code table [11].

When mechanistics of evolution of alternate genetic codes are investigated, it appears that almost all of the present-day alternate codes are much “better” than any random genetic code table constructed [4], although in their analyses of how reassignments can have occurred the researchers conclude that the “canonical” or standard genetic code is slightly better than the alternates. If one assumes error-minimization as the basic premise of optimality of code tables, then the standard genetic code appears to be the most optimized, as well as most adaptive when compared to alternative genetic codes [11]. In our study we observe that the Standard code is not the best among the alternate codes tested, in terms of robustness and toleration of mutations. This is probably due to our calculations taking into account only robustness of the genetic codes – however one study indicates that changeability of sequences is just as important for the evolution of genetic codes, thus adaptability, where the alternate genetic codes would suffer in our calculations [11, 12]: although seemingly contradictory, in this work robustness and changeability was implied to be equally fundamental for the survivability of organisms and evolution of genetic codes. Changeability is defined as a measure of how much a sequence can be altered through single base mutations [12], which, while putting a certain population of organisms at a certain disadvantage, could also lead to a slight advantage in another subset of organisms. This would be one explanation as to why the Standard Code, although so poor in terms of robustness, is the most widely-used coding table of all. As other researchers point out, although contemporary genomes operate in almost error-free environments, ancestors of the standard code were most likely in a highly error-prone niche, where robustness would have held a certain disadvantage [11]. A different explanation, in the light of still ongoing codon reassignments, could equally well be that the genetic codes are still changing and even possibly, evolving. Our hypothesis is more in line with the latter explanation, where the standard code was quite possibly the first optimal scheme reached by natural selection, but that it is still evolving, both reassigning certain codons, as well as expanding the amount of information contained by the table to the optimal number calculated for this coding table, 22 [5,7,8,9,10]. However such hypotheses need to be further tested after additional parameters such as the changeability have been included in the calculations. Also, combinatorics approaches could be utilized in order to provide comparison to the results obtained through the calculations presented in this study.

## 4. Methods

If there is a mutation on a gene which causes a severe change in the amino acid chain, we could safely assume that the organism would not be able to build a functional protein. If this protein is a crucial protein for the viability of the organism, one could also assume that any deleterious mutations in this gene rendering the protein non-functional would be lethal to the organism. Proteins taking part in immunity, cell respiration, DNA, RNA synthesis and cell division (tubulin formation) are basic examples for such crucial proteins. This has been previously reported to be the case for a human cytokine gene used in simulations and statistical calculations, following experimental findings in literature [8]. If all other effects (aging, food restriction, illness etc.) that are not directly related to the genetic code table are neglected, deleterious mutations changing the amino acid sequence will be the major cause of death in this *in silico* population. Neutral or “silent” mutations do not cause a change in viability in this model (see probability calculations below). We have also omitted reproduction from this model for simplicity, therefore we have a population which can only decrease as a result of deleterious mutations in order to emphasize the effects of coding tables alone.

There are many different schemes on the occurrence of mutations – in this preliminary model we have assumed that a mutation is a random process, with equal probability [13]. We disregard frameshift mutations in this particular model (caused by deletions or insertions), and we only look at single nucleotide substitutions – hence we include in this model the effects of only silent mutations, nonsense mutations and missense mutations on the protein product. Normally the rates for these replacements depend on the two nucleotides being interchanged. The simplest approach to the problem is to take all mutation rates to be equal, an approach known as the Jukes-Cantor mutation scheme [14].

The mutation is taken to be deleterious if it causes a change in the amino acid chain; and not all the mutations kill the individual. To be more explicit, the codons AAA and AAG in the Standard Genetic code the same amino acid, “lysine”; hence if AAA turns into AAG as a result of a mutation the amino acid will not change and the protein can be constructed safely. However; if AAA turns into AGA, which codes the amino acid “arginine”', the amino acid chain will change and we assume that the protein can not build up, which means the represented organism will die.

There can be a mutation which converts AAA to AAX where X ≠ {A, G, C, or T}; then the individual dies automatically. As a model, we are looking at a simpler case where a mutation changes A to one of G, C, or T, but not X. Since reproduction is not included in the model, the population can only diminish. The decrease in population can be found by calculating the probability of a deleterious mutation. The details of these calculations can be found in [8]. Essentially, the probability of the mutation changing the amino acid depends on the codon; so one needs to find the probability of hitting each different codon type. First, the probability of hitting a codon type (*P*_α_) is calculated as the ratio of the number of codons of that type in the gene (*N*_α_) to total number of codons. Then we need to exclude the mutations that do not cause a change in the amino acid and calculate the probability of a change occurring in the amino acid caused by a change in one nucleotide (*P*(*d*/α)). The results are reported as the negative of the slope, hence the smaller numbers indicate better survival rates over many generations. In general, we let the populations continue over a minimum of 10 generations.

We used only the exon (protein coding) part of the gene considering any mutation in the intron would be essentially harmless with respect to amino acid substitutions. As a simple example, the human cytokine gene has a total length of 2068 nucleotides; 621 nucleotides in exon part and 1447 ones in intron. The probability of hitting the exon part of the gene is simply the ratio of the exon part to the total gene:

(1) $$\text{P(hitting exon)}=\frac{621}{2068}=0.3032$$

Hence; the probability of having a *deleterious* mutation for all the gene is simply a product of mutation probability and probability of hitting the exon part of gene. As the chances of hitting any part of the gene is a same, we can neglect the intron part in the simulation since this would only be a multiplicative constant in the problem. Therefore the probability of having a deleterious mutation for all human cytokine gene is simply:

(2) $$\text{P(deleterious)}\propto \sum_{\alpha =1}^{64}P_{\alpha }P(d/\alpha )=0.7729.$$

where d is the number of deleterious mutations and α is the number of all possible mutations. Therefore, for our purposes, we have not used genomic sequences but rather CDS, or coding sequences, for the sake of simplicity because of the calculations discussed above.

The survival probability can be calculated by:

(3) P(surviving)=1-  P(deleterious)=0.2271

If we take an initial population of *N_0_* genes (individuals), after *n* number of mutations, to the first order, the number of surviving individuals (*N_n_*) is given by:

(4) $$N_{n}\approx N_{0}P{\left(\text{surviving}\right)}^{n}$$

Hence, we obtain the “probability of survival” with the slope of the number of surviving individuals versus time graph:

(5) slope≈ln[P(surviving)]=-1.4823

Similarly the probability of survival can be calculated for all the genes separately. However in this calculation once we make a change in the gene sequence and if the individual survives, we forget about the change we have made and restart the process for the second mutation cycle with the original gene sequence. We have assumed that in Nature, if the individual survives, the second mutation cycle starts with the mutated gene sequence and not the original one. Therefore, to be able to get closer to Nature we have also written a simulation code which allows for the mutation in the gene sequence to be kept in the next mutation mutation cycle.

The Standard Genetic Coding Table is shown in Figure 1. The variations of the different Coding Tables when compared to the Standard Coding Table are summarized in Table 1. The genes that have been used for this study are summarized in Table 2.

## Figures and Tables

**Table 1.** Comparison of various genetic coding tables (information accessed from NCBI Entrez).

Abbrev. Standard	Code Table Standard Code	Differences from Standard
VMC	The Vertebrate Mitochondrial Code	Code 2 Std
		AGA stop * Arg R
		AGG stop * Arg R
		AUA Met M Ile I
		UGA Trp W stop *
YMC	The Yeast Mitochondrial Code	Code 3 Std
		AUA Met M Ile I
		CUU Thr T Leu L
		CUC Thr T Leu L
		CUA Thr T Leu L
		CUG Thr T Leu L
		UGA Trp W stop *
		CGA absent Arg R
		CGC absent Arg R
MSC	The Mold, Protozoan, and Coelenterate Mitochondrial Code and the Mycoplasma/Spiroplasma Code	Code 4 Std
		UGA Trp W stop *
IMC	The Invertebrate Mitochondrial Code	Code 5 Std
		AGA Ser S Arg R
		AGG Ser S Arg R
		AUA Met M Ile I
		UGA Trp W stop *
CDH	The Ciliate, Dasycladacean and Hexamita Nuclear Code	Code 6 Std
		UAA Gln Q stop *
		UAG Gln Q stop *
EMC	The Echinoderm Mitochondrial Code	Code 9 Std
		AAA Asn N Lys K
		AGA Ser S Arg R
		AGG Ser S Arg R
		UGA Trp W stop *
ENC	The Euplotid Nuclear Code	Code 10 Std
		UGA Cys C stop *
AYNC	The Alternative Yeast Nuclear Code	Code 12 Std
		CUG Ser S Leu L
AMC	The Ascidian Mitochondrial Code	Code 13 Std
		AGA Gly G Arg R
		AGG Gly G Arg R
		AUA Met M Ile I
		UGA Trp W stop *
FMC	The Flatworm Mitochondrial Code	Code 14 Std
		AAA Asn N Lys K
		AGA Ser S Arg R
		AGG Ser S Arg R
		UAA Tyr Y stop *
		UGA Trp W stop *
BNC	Blepharisma Nuclear Code	Code 10 Std
		UAG Gln Q stop *

**Table 2.** Representative genes that are encoded by different coding tables were used in this study.

Coding Table	GenBank Accession Number	Gene Name / Explanation
YMC	X69431	*Kluyvermomyces thermotolerans* cox 2
YMC	X69430	*Candida glabrata* cox 2
YMC	X02439	*Hansenula saturnus* cox 2
YMC	AF442220	*Kluyveromyces lodderae* cox 2 (truncated)
YMC	KLU75348	*Kluyveromyces lactis* ATPase 9
YMC		Mitochondrion *Candida glabrata* average *
YMC		Mitochondrion *Kluyveromyces thermotolerans* average *
YMC		Mitochondrion *Kluyveromyces lactis* average *
IMC	AF329059; CDS 34-618	*Haementeria tuberculifera* NADH dehydrogenase subunit I (ND1) gene, partial cds; mitochondrial.
IMC	DQ202128;	*Drosophila stalkeri voucher* NADH dehydrogenase subunit 2
	CDS 32-520	(NADH2) gene, partial cds; mitochondrial.
IMC	AB275882	*Caenorhabditis* mitochondrial ND5 gene for NADH dehydrogenase
IMC	X99667	*Drosophila melanogaster* mRNA for mitochondrial ATPase synthase, subunit d
IMC	DROMTM2A	*Drosophila melanogaster* NADH dehydrogenase 3
IMC	AF164587	*Drosophila melanogaster* NADH dehydrogenase subunit 1
IMC	S76764	*Drosophila melanogaster* ND5, NADH dehydrogenase subunit 5
IMC		Caenorhabditis *elegans* average *
IMC		Drosophila *melanogaster* average *
VMC	NM_002488	*Homo sapiens* NADH hydrogenase 1 alpha subcomplex 2
VMC	BC128726	*Rattus norvegicus* ATP synthase, H+transporting, mitochondrial F0 complex, subunit c
VMC	BC010318	*Mus musculus* PEP carboxykinase 2, mitochondrial
VMC	X79547	*Equus caballus* mitochondrial DNA complete sequence NADH dehydrogenase
VMC	NM_001079924	*Pan troglodytes* NADH drhydrogenase (ubiquinone) 1 alpha subcomplex, NDUFA4, mitochondrial
VMC	PTU12706	*Pan troglodytes* Ptr5 mitochondrion cytochrome oxidase subunit II (COII) gene
VMC	NM_008617	*Mus musculus* malate dehydrogenase 2, NAD (mitochondrial) (Mdh2)
VMC	NM_029696	*Mus musculus* malate dehydrogenase 1B, NAD (soluble) (Mdh1b), mRNA, mitochondrial
VMC	NM_008618	*Mus musculus* malate dehydrogenase 1, NAD (soluble) (Mdh1)
VMC	NM_010344	*Mus musculus* glutathione reductase 1 (Gsr)
VMC	NM_001009329	*Felis catus* cytosolic malate dehydrogenase (MDH)
VMC		*Equus caballus* mitochondrion average *
VMC		*Pan troglodytes* mitochondrion average *
VMC		*Mus musculus* mitochondrion average *
FMC	AJ621238	*Echinococcus granulosus* malate dehydrogenase
FMC	AF188122	*Clonorchis sinensis* cytochrome oxidase subunit 1
FMC	DQ402037	*Echinococcus granulosus* NADH dehydrogenase subunit 1 (ND1) gene, partial cds; mitochondrial.
FMC	AY147416	*Echinococcus granulosus* thioredoxin glutathione reductase
FMC		*Flatworm* (*E. granulosus*) mitochondria average*
ENC	AY124990	*Euplotes aediculatus* alpha-2 platein precursor, gene, complete cds
ENC	X71353	*Euplotes octocarinatus* gamma tubulin
ENC	EF030059	*Euplotes nobilii* pheromone En-6
ENC	DQ866998	*Euplotes nobilii* heat shock protein 70
ENC	Y09551	*Euplotidae crassus* gamma tubulin 2
ENC	AF273753	*Euplates vannus* actin1
ENC	AY295877	*Euplates focardii* HSP70
ENC	DQ864704	*Euplotes octocarinatus* beta2 tubulin
ENC	S72098	*Euplates focardii* beta tubulin
ENC	J04533	*Euplotidae crassus* actin
ENC		*Euplotes focardii* average *
ENC		*Euplotes vannus* average *
CDH	AY293806	*Paraurostyla weissei* macronuclear DNA polymerase alpha gene, complete cds
CDH	HIU37081	*Hexamita inflata* elongation factor 1 alpha gene, partial cds.
CDH	Z11836	*Stylonychia lemnae* gene for DNA Polymerase II.
CDH	AY008386	*Urostyla grandis* macronuclear type II DNA polymerase alpha gene, complete cds.
CDH	X57926	*Stylonychia lemnae* EF1
CDH	AF194336	*Stylonychia lemnae* micronuclear DNA polymerase
CDH	XM_001032213	*Tetrahymena thermophila* EF1
CDH	XM_001031057	*Tetrahymena thermophila* EFG
CDH		*Tetrahymena thermophila* average *
CDH		*Stylonychia lemnae* average *
MSC	X65223	*Trichophyton rubrum* NADH 4L
MSC	X65223	*Trichophyton rubrum* cox 2
MSC	X65223	*Trichophyton rubrum* cox 1
MSC	NEUMTCOIJ	*Neurospora crassa* cox 2
MSC	AY548157	*Neurospora crassa* NADH dehydrogenase 1
MSC		*Neurospora crassa* average *
MSC		*Trichophyton rubrum* average *
Std	NM_001614	Human actin, gamma1
Std	AB062393	Human tubulin-beta
Std	AF141347	Human tubulin-alpha
Std	HUMACTA1	Human actin-beta
Std	AB292109	*Equus caballus* HSP70A8
Std	AB292108	*Equus caballus* EF1A1
Std	NM_001081838	*Equus caballus* actin beta
Std	X69884	*Equus caballus* CD2
Std	NM_001009165	*Pan troglodytes* EF1 alpha1
Std	NM_001009945	*Pan troglodytes* actin beta
Std	NM_001034095	*Pan troglodytes* tubulin alpha 1b
Std	NM_001045509	*Pan troglodytes* tubulin
Std	NM_001098544	*Pan troglodytes* tubulin alpha 1a
Std	NM_001098572	*Pan troglodytes* alpha 1
Std	AF091101	*Mus musculus* dUTPase
Std	MUSHSC70T	*Mus musculus* Hsc70T
Std	NM_007906	*Mus musculus* EF1 alpha 2
Std	NM_007393	*Mus musculus* actin beta
Std	NM_009609	*Mus musculus* actin gamma1
Std	NM_134024	*Mus musculus* tubulin gamma 1
Std	NM_009984	*Mus musculus* cathepsin L
Std	NM_013486	*Mus musculus* CD2
Std	NM_001009326	*Felis catus* EF1 alpha
Std	NM_001009841	*Felis catus* CD2
Std	EF407948	*Fasciola hepatica* cathepsin L mRNA (flatworm)
Std	EF201934	*Taenia asiatica* calcineurin B (flatworm)
Std	DQ256465	*Schistosoma mansoni* cathepsin-like protein CD2 (flatworm)
Std	EF199625	*Taenia solium* dUTPase (flatworm)
Std		Human average *
Std		*Equus caballus* average *
Std		*Pan troglodytes* average *
Std		*Mus musculus* average *
Std		*Felis catus* average *

* (based on genome-based codon usage frequencies obtained from http://www.kazusa.or.jp/codon/)
